# A Pine Enhanced Biochar Does Not Decrease Enteric CH_4_ Emissions, but Alters the Rumen Microbiota

**DOI:** 10.3389/fvets.2019.00308

**Published:** 2019-09-17

**Authors:** Stephanie A. Terry, Gabriel O. Ribeiro, Robert J. Gruninger, Alex V. Chaves, Karen A. Beauchemin, Erasmus Okine, Tim A. McAllister

**Affiliations:** ^1^Lethbridge Research and Development Centre, Agriculture and Agri-Food Canada, Lethbridge, AB, Canada; ^2^School of Life and Environmental Sciences, The University of Sydney, Sydney, NSW, Australia; ^3^Department of Production Animal Health, University of Calgary, Calgary, AB, Canada; ^4^Department of Biological Sciences, University of Lethbridge, Lethbridge, AB, Canada

**Keywords:** biochar, enteric methane, microbiome, 16s rRNA, rumen

## Abstract

The objective of this study was to examine the effect of a pine enhanced biochar (EB) on rumen fermentation, apparent total tract digestibility, methane (CH_4_) emissions, and the rumen and fecal microbiome of Angus × Hereford heifers fed a barley silage-based diet. The experiment was a replicated 4 × 4 Latin square using 8 ruminally cannulated heifers (565 ± 35 kg initial BW). The basal diet contained 60% barley silage, 35% barley grain and 5% mineral supplement with EB added at 0% (control), 0.5, 1.0, or 2.0% (DM basis). Each period lasted 28 days, consisting of 14 days adaptation and 14 days of measurements. Samples for profiling of the microbiome in rumen liquid, solids and feces were collected on d 15 before feeding. Rumen samples for fermentation characterization were taken at 0, 3, 6, and 12 h post feeding. Total collection of urine and feces was conducted from days 18 to 22. Heifers were housed in open-circuit respiratory chambers on days 26–28 to estimate CH_4_ emissions. Ruminal pH was recorded at 1-min intervals during CH_4_ measurements using indwelling pH loggers. Data were analyzed with the fixed effects of dietary treatment and random effects of square, heifer within square and period. Dry matter intake was similar across treatments (*P* = 0.21). Ammonia N concentration and protozoa counts responded quadratically (*P* = 0.01) to EB in which both were decreased by EB included at 0.5 and 1.0%, compared to the control and 2.0% EB. Minimum pH was increased (*P* = 0.04), and variation of pH was decreased (*P* = 0.03) by 2.0% EB. Total tract digestibility, N balance and CH_4_ production were not affected (*P* ≥ 0.17) by EB. Enhanced biochar decreased the relative abundance of *Fibrobacter* (*P* = 0.05) and *Tenericutes* (*P* = 0.01), and increased the relative abundance of *Spirochaetaes* (*P* = 0.01), *Verrucomicrobia* (*P* = 0.02), and *Elusimicrobia* (*P* = 0.02). Results suggest that at the examined concentrations, EB was ineffective at decreasing enteric CH_4_ emissions, but did alter specific rumen microbiota.

## Introduction

Ruminant production is a well-known contributor to global methane (CH_4_) emissions though it also plays an important role in meeting global protein demands. Dietary manipulation is one of the most successful strategies of mitigating enteric CH_4_ in ruminants. With pressure to reduce the carbon footprint of beef production, technologies that can simultaneously increase productive performance and decrease CH_4_ production are needed. Additionally, increasing production without affecting enteric CH_4_ fluxes can reduce CH_4_ emission intensity.

Enhanced biochar (EB) is a pyrolysed form of charcoal and has been postulated to concurrently enhance feed degradability and decrease enteric CH_4_ production (1). It is obtained by heating (350–600°C) plant biomass under oxygen (O_2_) limited conditions, resulting in a recalcitrant form of C (2) mixed with other inorganic nutrients (3).

Due to its large surface area, porous structure and high mineral content, EB has been utilized as an air and water scrubber, as well as a soil amendment (2, 4). Improvements in crop yields and soil fertility (5) as well as decreases in nitrous oxide (N_2_O) and CH_4_ from fields have been reported when biochar was included as a soil amendment (6, 7). Reports of positive ruminal responses to EB also exist (1, 8), with the hypothesis that the porous nature of biochar may be beneficial to the ruminal environment, promoting the formation of microbial biofilms which can result in an increase in feed degradation (8, 9). However, a promotion in microbial biofilm development could also promote the growth of the archaea responsible for ruminal CH_4_ production. Alternatively, Winders et al. (10) found that biochar had no effect on intake, digestibility of nutrients, or CH_4_ production in beef cattle.

Whilst the use of biochar in the diet has been examined both *in vitro* and *in vivo*, there is a lack of comprehensive research on the effects of biochar inclusion in the diet of beef cattle. Therefore, the objective of this study was to investigate the effects of a pine EB added to a barley silage-based diet on rumen fermentation, CH_4_ production, nutrient utilization and the rumen and fecal microbiota in beef heifers.

## Materials and Methods

This study was conducted at the Agriculture and Agri-Food Canada Research and Development Centre in Lethbridge, Alberta, Canada. The heifers were cared for in accordance with the guidelines of the Canadian Council on Animal Care (11). The study procedures for animal use were reviewed and approved by the institutional Animal Care Committee at the research center (ACC1726).

### Experiment Design

This experiment was conducted as a replicated 4 × 4 Latin square using 4 heifers per square, with four 28-days periods, and 4 dietary treatments. Eight ruminally cannulated Angus heifers (565 ± 36 kg) were blocked by weight and randomly assigned to a square and treatment sequence. The first 14 days of each period were used for dietary adaptation with the remaining 14 days used for measurements and sample collection. The period for each square was staggered by a week to accommodate the availability of the open-circuit respiratory chambers. Heifers were housed in tie-stalls in a metabolism barn and given exercise daily, except for when samples were being collected.

### Dietary Treatments

Heifers were offered a basal diet of 60% barley silage, 35% barley grain and 5% mineral supplement (DM basis; Table 1). Dietary treatments consisted of adding EB at 0, 0.5, 1.0, and 2.0% of dietary DM (Cool Planet, CO, USA) to the basal diet. The basal diet was formulated as a typical Canadian beef feedlot backgrounding diet meeting the requirements of beef heifers according to NASEM (12).

**Table 1:** Chemical composition of dietary components and the total mixed ration (TMR) fed to heifers.

**Component**	**TMR**	**Barley silage**	**Barley grain**	**Mineral** **supplement**	**EB^a^**
Ingredients, % DM		60.0	35.0	5.0	
Composition, % DM					
DM	48.0	37.4	93.6	95.4	92.6
OM	93.6	92.9	97.7	68.2	98.8
CP	12.0	10.7	13.8	18.0	
NDF	30.3	40.1	17.1	16.5	61.6
ADF	14.0	20.9	3.7	4.0	52.5
Starch	34.4	22.3	57.3	29.1	
GE, Mcal/kg	4.49	4.61	4.73	3.32	2.05

a *EB, enhanced biochar*.

The EB was supplied by Cool Planet Energy Systems, Inc. (Greenwood Village, CO, USA) which markets biochar products under brand names, Cool Terra® and Cool Fauna®. The biomass used for the EB was from southern yellow pine from the United States. The EB provided was produced using the company's proprietary Engineered Biocarbon™ Technology, which includes front-end pyrolysis (below 650°C for several minutes) and a patented post-pyrolysis treatment step. The post-treated biochar was ground to a consistent particle size. The final product underwent a comprehensive chemical analysis (Table 2) and had a dioxin content less than the EU maximum limit (<0.75 ng/kg).

**Table 2:** Chemical and physical characteristics of enhanced biochar.

**Component**	**Composition**
Carbon, % DM	75
pH	7–8
Bulk density, kg/m^3^	160–256
Surface area, m^2^/g	200–250
Porosity, vol%	60–70
Water holding capacity, wt%	150–250
**Particle size distribution**	
< 0.5 mm, % DM	80
0.5–2.0 mm, % DM	20

### Feed Sampling and Intake

Heifers were fed at *ad libitum* intake (5% orts) during the adaptation period and were restricted to 90% of intake during measurement of total tract digestibility and CH_4_. The EB was top dressed onto the diet, and mixed manually into the total mixed ration (TMR). Heifers were fed at 0900 h once daily. Samples of barley grain and mineral supplement were collected each period with barley silage and the TMR sampled weekly to determine DM. If silage DM deviated by more than ±3 units from the average, the TMR was adjusted accordingly.

During weeks of total tract digestibility or chamber measurements, daily samples of silage and TMR were sampled and then pooled by week. Feed refusals were weighed daily, sampled, and then pooled by week. All feed samples were dried at 55°C for 72 h for determination of DM.

### Rumen Fermentation and Microbiome

Rumen samples (~300 g) were taken on d 15 from each heifer before feeding, then 3, 6, and 12 h post feeding. A liquid sample (40 mL) was taken from the liquid phase of the rumen using a syringe vacuum for dissolved H_2_ analysis. Four subsequent rumen content samples were taken from the reticulum, and the ventral, caudal and dorsal-ventral sacs within the rumen and pooled. Rumen contents were then squeezed through 2 layers of PECAP nylon (pore size 355 μm; Sefar Canada Inc., Ville St. Laurent, Canada) to separate the solid and liquid components. A subsample of solid rumen contents (30 g) and the liquid filtrate (40 mL) were flash frozen in liquid N for microbial profiling to quantify solid associated (SAM) and liquid associated microbes (LAM), respectively. Fecal samples from each heifer were taken before feeding for microbial profiling and flash frozen in liquid N to quantify fecal associated microbes (FAM). Only samples taken before feeding (0 h) were used for microbial profiling and were stored at −80°C until further processing.

At each time point, the pH of the filtrate was determined (not reported; Orion model 260A, Fisher Scientific, Toronto, ON) and two, 5 mL samples were placed in prefilled vials containing either 1 mL of 25% (wt/vol) metaphosphoric acid or 1 mL of 1% (vol/vol) sulfuric acid for analysis of volatile fatty acids (VFA) and ammonia-N (NH_3_-N), respectively. Vials were stored at −20°C until analyzed. A further 5 mL of filtrate was taken for protozoa enumeration and placed in a 12 mL scintillation vial prefilled with 5 mL of methyl-green-formalin-saline solution.

### Apparent Total Tract Digestibility

For determination of apparent total tract digestibility of nutrients, total urine and fecal collection was conducted for four, 24 h periods from days 18 to 22 of each experimental period. Heifers were housed in individual collection tie-stalls and were fitted with indwelling urinary catheter balloons (Bardex Lubricath Foley catheter, Bard Canada Inc., Oakville, ON) to avoid cross-contamination of urine and feces. Feces were collected in a pan placed behind each heifer. A 10% fecal subsample from each heifer was taken daily and composited by heifer within period. A representative sample (500 g) was dried at 55°C for 96 h for determination of DM. Urine was collected in a closed collection container prefilled with 4 *N* H_2_SO_4_ (500 mL) to prevent volatilization of NH_3_-N. A 2% acidified urine sample was taken daily and then composited by heifer within period. The urine subsamples were diluted with distilled water at a ratio of 1:5 and stored at −20°C until analyzed.

### Blood Sampling

On d 22, blood samples were taken from heifers before feeding and 6 h after feeding from the jugular vein. Blood was taken using two 10 mL vacuum tubes containing lithium heparin (Vacutainer; Becton Dickinson, Mississauga, Canada) for plasma urea N (**PUN**) analysis. Tubes were centrifuged at 2,000 × g for 20 min at 4°C, then plasma was transferred to microtubes and frozen at −20°C until analyzed.

### Ruminal pH Loggers

On d 25, a pH logger was placed into the ventral sac of each heifer for determination of rumen pH. Ruminal pH was recorded using the LRCpH data logger system [Dascor, Escondido, CA; (13)] with pH measurements recorded at 1 min intervals from d 25 to 27. The loggers were standardized using pH 4 and 7 buffers before and after their use.

### Chamber Measurements

For measurement of CH_4_ emissions, heifers were housed in 4 separate open-circuit respiratory chambers (4.4 m wide × 3.7 m deep × 3.9 m tall, 63.5 m^3^ volume, model C1330; Conviron Inc., Winnipeg, MB). On day 26, heifers were moved to a controlled environment building and led into their allocated chamber where they were immediately fed and the chamber doors were closed. Heifers were housed in the chambers for three, 24 h periods with the chambers opened once daily for feeding and cleaning. A detailed description of methodology and emission calculations is provided by Beauchemin and McGinn (14). Chambers were calibrated before and after the commencement of the study, with the instruments calibrated daily (14). Variability in slopes across chambers was less than 5% and recovery rates ranged from 98% to 106%.

### Chemical Analysis and Calculations

Feed, feed refusals and fecal samples were dried at 55°C for determination of DM and subsequently ground through a 1 mm screen (Standard model 4 Wiley mill; Arthur H. Thomas, Philadelphia, PA). Samples were analyzed for analytical DM [(15); method 930.15], OM (method 942.05), ash (method 942.05), neutral detergent fiber (NDF), and acid detergent fiber (ADF). Ash content was determined by combustion of samples in a muffle furnace at 550°C for 5 h. Samples were analyzed sequentially for NDF (16) and ADF [(15); method 973.18] with modifications for using a fiber analyzer [F57 Fiber Filter Bags, 200 Fiber Analyzer, ANKOM Technology; (17)] with heat-stable α amylase (Termamyl 120, Sigma-Aldrich, St. Louis, MO) and sodium sulfite included in the NDF procedure, with NDF expressed exclusive of residual ash.

For determination of starch and N content, subsamples of dried feed and feces were ground in a ball mill (Mixer Mill MM2000, Retsch, Haan, Germany). For urinary N, 100 μL of diluted urine was oven dried at 55°C for 24 h. Nitrogen in feed, refused feed, feces, and urine was quantified by flash combustion with gas chromatography and thermal conductivity detection [Carlo Erba Instruments, Milan, Italy; (15); method 990.03]. Crude protein (CP) was calculated as N × 6.25. Starch was determined as described by Herrera-Saldana et al. (18), by hydrolyzing α-glucose polymers using a mixture of amyloglucosidase (Megazyme International Ltd., Wicklow Ireland) and 1,4-α-D-glucan glucanohydrolase (Brennfag Canada Inc., Toronto, ON). Absorbance of glucose samples was read on a Thermo Scientific Appliskan 1.437 (SkanIt Software 2.3 RE) microplate reader at a wavelength of 490 nm. Gross energy content of feed and fecal samples was determined using a bomb calorimeter (model E2k, CAL2k, Johannesburg, South Africa).

Dissolved H_2_ was measured using a sensor (H_2_-500; Unisense, Aarhus, Denmark) attached to a glass flow-through cell (2 mm internal diameter, 6 mm external diameter). The H_2_ sensor was connected to a microsensor multimeter (Unisense, Aarhus, Denmark). Calibration of the sensor was as described by Guyader et al. (19). Protozoa were enumerated as described by Dehority (20) under a light microscope using a Levy-Hausser counting chamber (Hausser Scientific, Horsham, PA) with 1-mm depth. Rumen VFA were determined using a gas chromatograph (5890A Series Plus II, Hewlett Packard Co., Palo Alto, CA) equipped with a 30-m Zebron free fatty acid phase fused silica capillary, 0.32-mm i.d., and 1.0-μm film thickness column (Phenomenex, Torrance, CA).

The concentration of NH_3_-N was determined by the phenol-hypochlorite method as described by Broderick and Kang (21). Plasma urea N concentration was determined using a microsegmented flow analyzer (model Astoria2; Astoria Pacific Inc., Clackamas, OR). Allantoin was determined as described by Young and Conway (22) and uric acid concentration was determined by a colorimetric method using a commercial kit (MAK077, Sigma-Aldrich Co., St. Louis, MO).

Microbial N synthesis was estimated using the calculation formulated by Chen and Gomes (23):

$$\begin{array}{l} \text{Microbial PD absorbed =}\\ \;\frac{\left(\text{Total PD excretion − 0}.\text{385 }\times \;{\text{BW}}^{\text{0}.\text{75}}\right)}{\text{0}.\text{85}} \end{array}$$

Where total purine derivatives (PD) is the sum of allantoin and uric acid (mmol/d) excreted. Consecutively, microbial flow (g N/d) was calculated as:

$$\text{Microbial N flow = }\frac{\text{PD absorbed }\times \text{ 70}}{\text{0}.\text{116 }\times \text{ 0}.\text{83 }\times \text{ 1000}}$$

Heat production (HP) was calculated using the equation proposed by Brouwer (24) using the average measurements of O_2_ consumed and CH_4_ and carbon dioxide (CO_2_) produced over 3 days of chamber measurements:

$$\begin{array}{l} \text{HP }\left(\text{kcal/d}\right)\text{ = 3}.\text{886 }\times \;O_{2}\left(\text{L/d}\right)\text{ + 1}.\text{2 }\times \;{CO}_{2}\left(\text{L/d}\right)\\ \text{ − 1}.\text{431 }\times \text{ urinary N}\left(\text{g/d}\right)\text{ − 0}.\text{518 }\times \;{\text{CH}}_{4}(\text{L/d}) \end{array}$$

### Rumen Microbial Community

Only samples from heifers on control or 2.0% EB treatment were used for microbial profiling. Samples were freeze dried and ground using a coffee grinder. DNA was extracted from ~0.1 g of the freeze dried, ground material using the Zymobiomics DNA extraction kit (Zymo Research, Irvine CA). Concentration and purity of the extracted metagenomic DNA was determined by measuring the ratios of absorbance at 260/280 and 260/230 using a NanoDrop spectrophotometer (Thermo Fisher Scientific, Mississauga, ON). A PCR reaction was conducted to amplify the full length 16s rRNA gene using the primers 27F (5′-AGAGTTTGATCMTGGCTCAG-3′) and 1398R (5′-TACGGYTACCTTGTTACGACTT-3′) to confirm that there were no PCR inhibitors present in the sample.

Sequencing was performed at McGill University and Genome Quebec Innovation Center (Montreal, Canada) using the Illumina MiSeq Reagent Kit v2 (500 cycle) following the manufacturer's guidelines. The primers 515F (5′-GTGCCAGCMGCCGCGGTAA-3′) and 806R (5′-GGACTACHVGGGTWTCTAAT-3′) targeting the V4 region of the 16s rRNA gene were used to examine both bacterial and archaeal diversity. A 33 cycle PCR using 1 μL of a 1 in 10 dilution of genomic DNA and the Fast Start High Fidelity PCR System (Roche, Montreal, PQ) was conducted with the following conditions: 94°C for 2 min, followed by 33 cycles of 94°C for 30 s, 58°C for 30 s, and 72°C for 30 s, with a final elongation step at 72°C for 7 min. Fluidigm Corporation (San Francisco, CA) barcodes were incorporated in a second PCR reaction using the FastStart High Fidelity PCR System under the following conditions: 95°C for 10 min, followed by 15 cycles of 95°C for 15 s, 60°C for 30 s, and 72°C for 1 min, followed by a final elongation step at 72°C for 3 min. After amplification, PCR products were assessed in a 2% agarose gel to confirm adequate amplification. All samples were quantified using the Quant-iT PicoGreen dsDNA Assay Kit (Life Technologies, Carlsbad, CA) and were pooled in equal proportions. Pooled samples were then purified using calibrated Ampure XP beads (Beckman Coulter, Mississauga, ON). The pooled samples (library) were quantified using the Quant-iT PicoGreen dsDNA Assay Kit (Life Technologies, Carlsbad, CA) and the Kapa Illumina GA with Revised Primers-SYBR Fast Universal kit (Kapa Biosystems, Wilmington, MA). Average fragment size was determined using a LabChip GX (PerkinElmer, Waltham, MA, USA) instrument.

Raw fastq files were imported into Qiime2 for sequence analysis. Primer and adapter sequences were removed from sequence files with the plugin cutadapt (25). Following removal of primer and adapter sequences the program dada2 (26) was used for quality control, filtering of any phiX reads present in the sequencing data and removal of chimeric sequences. The Dada2 model was used to correct errors in Illumina sequence data and generate a feature table containing count data (abundance) of sequences at strain level resolution (>99.9% id OTUs) (26). Following Dada2, the Mafft program was used to perform a multiple sequence alignment and to mask highly variable regions. A phylogenetic tree was generated with FastTree (27). Taxonomy was assigned to sequences using a Naïve-Bayes classifier trained with the Silva 128 reference database and the feature-classifier plugin (28). Samples were subsampled to the lowest number of sequences found in all samples to ensure that α- and β-diversity analysis used the same number of sequences per sample. The diversity plugin core-diversity-metrics was used to asses microbial diversity within (α-diversity) and between samples (β-diversity). α-Diversity (Shannon's diversity index), phylogenetic diversity (Faith's phylogenetic diversity), number of observed OTU, evenness (Pielou's Evenness) and taxonomic abundance were evaluated. β-Diversity analysis was carried out using weighted and unweighted UniFrac (29). Sequences were deposited to the Small Reads Archive (NCBI) with accession number PRJNA534330.

### Statistical Analysis

Data were analyzed using the MIXED model procedure of SAS (SAS Institute Inc., Cary, NC). The univariate procedure was used to verify that data were normally distributed. Protozoal counts were log_10_ transformed before statistical analysis to conform to the homogeneity of variance. Data were analyzed with heifer as experimental unit for all variables, except for CH_4_ measurements where heifer within chamber was the experimental unit as each heifer was kept within the same chamber for each period. The model included EB as a fixed effect with random effects of square, heifer nested within square, and period nested within square. For microbial profiling, the fixed effects of source (LAM, SAM, FAM) and treatment × source interaction were also included. Hour was treated as a repeated measure for rumen variables and blood parameters, with day as a repeated measure for chamber and pH logger measurements. Continuous ruminal pH data were summarized for daily average, minimum, maximum and standard deviation using SAS (SAS Institute Inc., Cary, NC). Cumulative daily CH_4_ emissions from each chamber was calculated for each of the 4 d within each period. The minimum value of Akaike's information criteria were used to select the appropriate covariance structure. False discovery rate (FDR)-corrected *P*-values were calculated using Tukey's test. Differences between means were declared when *P* < 0.05, and contrast statements were used to test for linear and quadratic effects using coefficients adjusted for non-equally spaced treatment structure. Chord diagrams were generated using the circlize package in R Studio (30).

## Results

Dry matter intake and total VFA concentration were similar across treatments (*P* > 0.21; Table 3). The concentration of acetate in heifers fed 0.5 and 2.0% EB was higher (*P* = 0.01) than those fed 1.0% EB. There was a quadratic tendency (*P* = 0.09) for isobutyrate concentration and dissolved H_2_ to decrease with increasing EB treatment. Ammonia-N concentration responded quadratically (*P* = 0.01) with 0.5% EB initially decreasing NH_3_-N concentrations, however there was no difference between the control and 1.0% and 2.0% EB.

**Table 3:** Dry matter intake and ruminal fermentation products of beef heifers fed a barley silage-based diet containing increasing addition of enhanced biochar (EB; *n* = 8 per treatment).

	**Treatment^1^**					***P***-value**^2^**		
**Item**	**Control**	**0.5% EB**	**1.0% EB**	**2.0% EB**	**SEM**	**Treat**	**L**	**Q**
DMI, kg/d	10.2	9.77	9.84	10.4	0.42	0.21	–	–
Total VFA, mM	134.6	126.1	123.6	133.3	5.628	0.23	–	–
**VFA, mol/100 mol**								
Acetate (A)	63.4^ab^	64.2^a^	62.2^b^	63.9^a^	0.92	0.01	–	–
Propionate (P)	18.8	18.3	19.7	18.6	0.87	0.51	–	–
Butyrate	13.1	12.8	13.2	13.0	0.69	0.85	–	–
Isobutyrate	0.87^a^	0.86^a^	0.88^a^	0.82^b^	0.026	0.02	0.07	0.09
Valerate	1.46	1.41	1.51	1.44	0.063	0.20	–	–
Isovalerate	1.56	1.53	1.67	1.50	0.087	0.40	–	–
Caproate	0.69	0.69	0.62	0.71	0.054	0.40	–	–
A:P ratio	3.43	3.59	3.26	3.49	0.194	0.41	–	–
NH_3_-N, mM	5.27^a^	3.86^b^	4.55^ab^	5.53^a^	1.141	0.02	–	0.01
Dissolved H_2_	102.7	84.4	72.8	99.5	18.75	0.08	–	–

1 *Control: no EB; 0.5% EB: EB added at 0.5% DM, 1.0% EB: EB added at 1.0% DM, 2.0% EB: EB added at 2.0% DM*.

2 *L, linear effect; Q, quadratic effect*. *Different lowercase letters in rows indicate significantly different means (P < 0.05)*.

Total protozoa counts decreased quadratically (*P* < 0.01) with total counts in the control being higher than all concentrations of EB (Table 4). The percentage of *Entodinium, Holotrichs* and other protozoal genera did not differ among treatments (*P* > 0.14). Minimum and variation of pH responded quadratically (*P* < 0.06) to increasing concentration of EB where 2.0% EB increased minimal pH and decreased pH variation.

**Table 4:** Protozoa counts and rumen pH of beef heifers fed a barley silage-based diet containing increasing addition of enhanced biochar (EB; *n* = 8 per treatment).

	**Treatment^1^**					***P***-value**^2^**		
**Item**	**Control**	**0.5% EB**	**1.0% EB**	**2.0% EB**	**SEM**	**Treat**	**L**	**Q**
Protozoa, n × 10^5^	8.37^a^	6.10^b^	5.68^b^	6.98^ab^	1.167	0.05	–	<0.01
Entodinium, %	87.4	87.1	85.8	84.1	5.35	0.14	–	–
Holotrichs^3^, %	4.37	3.56	4.45	4.66	0.819	0.21	–	–
Other^4^, %	4.19	4.64	5.15	6.10	1.454	0.27	–	–
**Ruminal pH**^**5**^								
Min	5.41^b^	5.35^b^	5.35^b^	5.61^a^	0.085	0.04	0.02	0.06
Mean	6.07	6.06	6.02	6.22	0.095	0.15	–	–
Max	6.81	6.88	6.76	6.84	0.079	0.44	–	–
SD	0.38^b^	0.45^a^	0.40^ab^	0.33^c^	0.028	0.03	0.04	0.03

1 *Control: no EB; 0.5% EB: EB added at 0.5% DM, 1.0% EB: EB added at 1.0% DM, 2.0% EB: EB added at 2.0% DM*.

2 *L, linear effect; Q, quadratic effect*.

3 *Holotrichs = Dasytricha + Isotricha*.

4 *Other = Entodinium + Polyplastron + Epidinium + Ophryoscolex + Metadinium + Ostracodinium + Eudiplodinium + Eremoplastron*.

5 *Ruminal pH was measured at 1 min intervals over 3 d using continuous pH loggers*. *Different lowercase letters in rows indicate significantly different means (P < 0.05)*.

The inclusion of EB had no effect (*P* > 0.17) on nutrient digestibility or nitrogen balance (Tables 5, 6). Total PD production, MP absorbed and MN flow were not altered (*P* = 0.22) by EB (Table 6). Energy balance, heat production and methane production (g/d, g/kg DMI, and g/kg DMD) were also not affected (*P* > 0.20) by EB (Tables 7, 8).

**Table 5:** Apparent total tract nutrient digestibility of beef heifers fed a barley silage-based diet containing increasing addition of enhanced biochar (EB; *n* = 8 per treatment).

	**Treatment^a^**					***P*-value^b^**
**Item**	**Control**	**0.5% EB**	**1.0% EB**	**2.0% EB**	**SEM**	**Treat**
**Digestibility, %**						
DM	70.0	68.2	68.5	68.3	0.96	0.20
OM	72.3	69.9	71.1	70.8	1.03	0.17
CP	58.9	57.4	57.6	58.4	1.70	0.72
NDF	50.0	47.9	48.3	48.6	2.93	0.72
ADF	50.7	46.6	46.7	47.2	3.39	0.36
Starch	94.5	94.2	95.2	94.2	1.09	0.67
GE	59.6	57.9	57.8	57.1	1.37	0.34

a *Control: no EB; 0.5% EB: EB added at 0.5% DM, 1.0% EB: EB added at 1.0% DM, 2.0% EB: EB added at 2.0% DM*.

b *L, linear effect; Q, quadratic effect*.

**Table 6:** Nitrogen balance of beef heifers fed a barley silage-based diet containing increasing addition of enhanced biochar (EB; *n* = 8 per treatment).

	**Treatment^a^**					***P*-value^b^**
**Item^c^**	**Control**	**0.5% EB**	**1.0% EB**	**2.0% EB**	**SEM**	**Treat**
N intake, g/d	195.4	186.2	187.5	194.6	9.54	0.38
Total N excretion, g/d	156.4	156.6	152.4	156.9	7.72	0.90
Total N retention, g/d	30.0	29.6	35.2	30.2	5.12	0.36
Plasma urea N, mg/L	79.6	72.4	76.4	79.3	6.34	0.32
Total PD, mmol/d	109.2	104.5	116.0	94.1	9.82	0.22
MP Absorbed^d^	70.8	65.6	79.1	53.1	11.28	0.22
MN Flow, g N/BW^0.75^/d	0.40	0.38	0.45	0.30	0.062	0.22

a *Control: no EB; 0.5% EB: EB added at 0.5% DM, 1.0% EB: EB added at 1.0% DM, 2.0% EB: EB added at 2.0% DM*.

b *L, linear effect; Q, quadratic effect*.

c *PD = purine derivatives; MP = microbial protein; MN = microbial nitrogen*.

d *Microbial N synthesis was estimated using measurements of allantoin and uric acid excreted in urine as described by Chen and Gomes (23)*.

**Table 7:** Energy balance of beef heifers fed a barley silage-based diet containing increasing addition of enhanced biochar (EB; *n* = 8 per treatment).

	**Treatment^a^**					***P*-value^b^**
**Item**	**Control**	**0.5% EB**	**1.0% EB**	**2.0% EB**	**SEM**	**Treat**
**Energy, Mcal/d^c^**						
GEI^d^	45.0	43.6	44.1	46.9	1.90	0.42
Feces	15.3	15.6	15.9	17.0	0.81	0.25
DE^e^	29.7	27.9	28.2	29.8	1.36	0.53
Methane	2.77	2.67	2.65	2.76	0.160	0.59
HP^f^	13.9	13.7	14.4	13.5	0.65	0.75
DE, Mcal/kg DM	2.94	2.87	2.92	2.88	0.082	0.92
**GEI, %**						
Feces	40.7	42.5	42.6	43.3	1.37	0.47
DE	59.3	57.5	57.4	56.7	1.37	0.47
Methane	6.02	6.02	5.88	5.76	0.286	0.42
ME^g^	51.7	49.8	49.8	49.3	1.56	0.43
HP	30.8	31.8	32.4	28.8	1.52	0.36
RE^h^	20.9	18.0	17.3	20.5	2.14	0.57

a *Control: no EB; 0.5% EB: EB added at 0.5% DM, 1.0% EB: EB added at 1.0% DM, 2.0% EB: EB added at 2.0% DM*.

b *L, linear effect; Q, quadratic effect*.

c *Energy, Mcal/d unless stated otherwise*.

d *GEI = gross energy intake = GE content (Mcal/kg DM) × DMI (kg/d)*.

e *DE = digestible energy = GEI (Mcal/d) – fecal energy (Mcal/d)*.

f *HP = heat production = as calculated by (24)*.

g *ME = metabolisable energy = 100 – fecal energy (%) – urinary energy (%) – methane energy (%)*.

h *RE = retained energy = ME (%) – HP(%)*.

**Table 8:** Methane emissions of beef heifers fed a barley silage-based diet containing increasing addition of enhanced biochar (EB; *n* = 8 per treatment).

	**Treatment^a^**					***P*-value^b^**
**CH_**4**_**	**Control**	**0.5% EB**	**1.0% EB**	**2.0% EB**	**SEM**	**Treat**
g/d	225.1	216.5	215.5	224.5	11.95	0.43
g/kg DMI	23.8	23.5	24.1	24.9	1.17	0.59
g/kg DMD	3.22	3.17	3.15	3.36	0.177	0.35
g/kg BW	0.35	0.34	0.34	0.36	0.022	0.20
kg CO_2_eq/d	5.72	5.51	5.48	5.71	0.329	0.61

a *Control: no EB; 0.5% EB: EB added at 0.5% DM, 1.0% EB: EB added at 1.0% DM, 2.0% EB: EB added at 2.0% DM*.

b *L, linear effect; Q, quadratic effect*.

**Figure 1:**
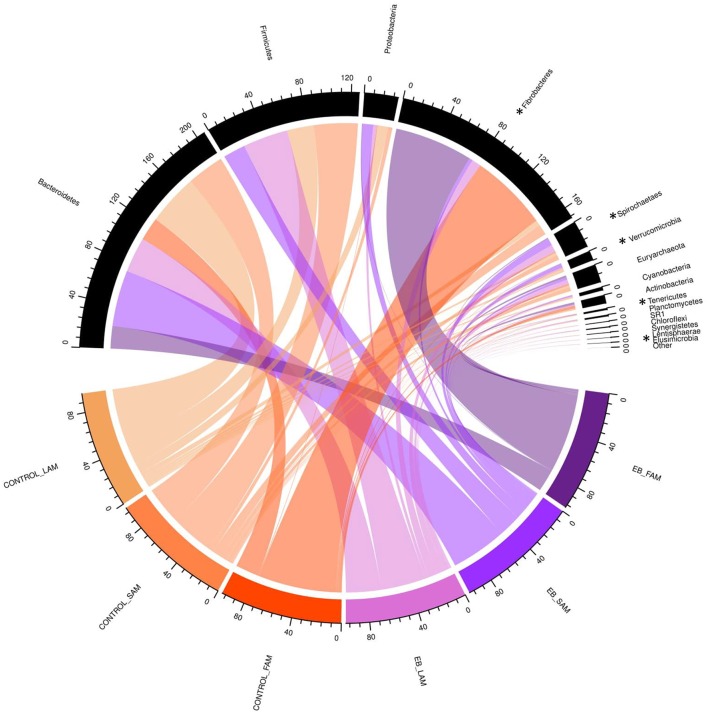
Chord diagram displaying the relative abundance (%) of phylum associated with treatment (Control and 2.0% enhanced biochar) and source (SAM, solid associated microbes; LAM, liquid associated microbes; FAM, fecal associated microbes). The scale indicates total accumulative abundance. The width of each chord represents the relative abundance of each phylum that is associated with each treatment and source. Phylum with ^*^ indicate those with significant differences.

**Figure 2:**
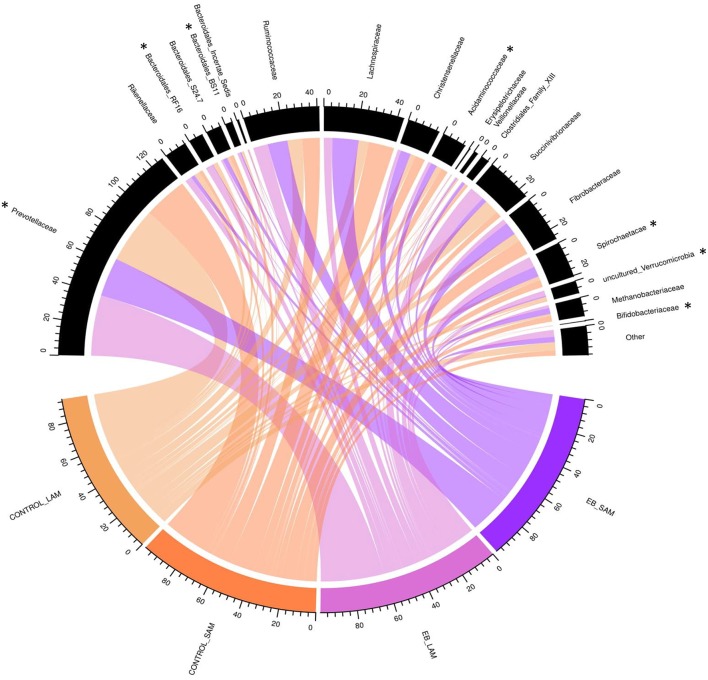
Chord diagram displaying the relative abundance (%) of families associated with treatment (Control and 2.0% enhanced biochar) and source (SAM, solid associated microbes; LAM, liquid associated microbes). The scale indicates total accumulative abundance. The width of each chord represents the relative abundance of each family that is associated with each treatment and source. Family with ^*^indicate those with significant differences.

Observed OTUs, measures of evenness, and diversity showed no difference (*P* ≥ 0.58) among EB treatments, but all differed (*P* < 0.01) with regard to origin of the sample (Table 9). There was an EB treatment by sample origin interaction (*P* < 0.05) with regard to the relative abundance of *Spirochaetaes, Verrucomicrobia, Tenericutes* and *Elusimicrobia*. *Spirochaetaes* were increased in LAM and SAM by EB (Figure 1). Only the relative abundance of *Verrucomicrobia* increased in LAM with EB. In LAM, EB decreased the relative abundance of *Tenericutes* and increased the relative abundance of *Elusimicrobia*. The relative abundance of *Fibrobacteres* was decreased (*P* = 0.05) by EB in both LAM and SAM. There was a EB by sample origin interaction (*P* < 0.05) with regard to the relative abundance of *Prevotellaceae, Bacteroidales* RF16 group, *Bacteroidales* BS11 gut group, *Spirochaetaceae*, and uncultured *Verrucomicrobia* (Figure 2). The relative abundance of *Prevotellaceae, Bacteroidales* RF16 group, *Bacteroidales* BS11 gut group and uncultured *Verrucomicrobia* were all increased in the LAM fraction by EB. *Spirochaetaceae* were increased by EB in both LAM and SAM by 63 and 30.3%, respectively. Enhanced biochar reduced (*P* < 0.05) the relative abundance of *Acidaminococcaceae* and *Bifidobacteriaceae* in both LAM and SAM fractions. For the FAM fraction, there was no effect of EB at the phylum or family level but there was a tendency (*P* = 0.06) for increased relative abundance of *Euryarchaeot*a with EB.

**Table 9:** α-diversity indices of liquid-(LAM), solid-(SAM) and fecal-(FAM) associated microbes of beef heifers fed a barley silage-based diet containing 2.0% enhanced biochar (EB; *n* = 8 per treatment).

	**LAM^a^**		**SAM**		**FAM**			***P*** **value**		
**Item**	**Control^b^**	**EB**	**Control**	**EB**	**Control**	**EB**	**SEM**	**Treat**	**Source^c^**	**Treat × Source**
Observed OTUs	908	879	1,040	1,041	733	736	37.0	0.76	<0.01	0.86
Simpsons evenness	0.80	0.80	0.83	0.83	0.81	0.81	0.008	0.93	<0.01	0.64
Phylogenetic diversity	52.1	52.1	53.6	54.8	35.3	35.9	1.45	0.58	<0.01	0.89
Shannon's diversity	7.93	7.83	8.36	8.33	7.56	7.74	0.119	0.87	<0.01	0.40

a *LAM, liquid associated microbes; SAM, solid associated microbes; FAM, fecal associated microbes*.

b *Control: no EB; EB: EB added at 2.0% DM*.

c *Source = LAM, SAM, FAM*.

## Discussion

Biochar has been proposed as a feed additive with the potential to enhance rumen fermentation and mitigate enteric CH_4_ production (8). This comprehensive study examined the effects of EB on beef cattle metabolism and microbial populations. To our knowledge, this is the first study to examine the effect of EB in rumen and fecal microbiota and CH_4_ emissions using 16S rRNA sequencing and open-circuit respiratory chambers, respectively.

One difficulty in assessing the effects of biochar on ruminal metabolism is the variability that can be introduced as a result of different sources of biomass, variation in the duration and temperature of pyrolysis as well as post-treatment modifications that can alter EB composition, porosity and chemistry. Although previous studies have found positive responses to a rice husk biochar (8), the present study utilized a pine EB due to its mass availability across the northern hemisphere. However, pine was the biomass used by both Saleem et al. (1) and Winders et al. (10) and the biochar still differed in surface area, bulk density and pH. These factors may affect the activity of EB within the rumen and subsequent metabolic responses in both the microbial population and the host animal.

Enhanced biochar had no effect on total VFA concentration, or their individual proportions compared to the control. This is in agreement with Calvelo Pereira et al. (31) who reported that although the original biomass (corn stover vs. pine) and inclusion level (81 vs. 186 g/kg DM) of biochar altered VFA proportions *in vitro*, these did not differ from the control treatment (silage, no biochar). McFarlane et al. (32) also found that different sources of biochar (i.e., yellow poplar, white pine and chestnut oak) did not alter VFA production *in vitro* but this experiment lacked a control. Using the rumen simulation technique (RUSITEC), Saleem et al. (1) found that total VFA, as well as acetate, propionate, butyrate and branched-chain VFA (BCVFA) production were linearly increased with increasing inclusion (up to 2.0% of diet DM) of EB in a barley silage-based diet. In the present study, only the branched-chain VFA isobutyrate, the by-product of valine, isoleucine, and leucine deamination (33) was decreased by 2.0% EB. The lack of change observed in iso-fatty acid concentration and MP synthesis is consistent with the relationship between deamination of amino acids and microbial nitrogen flow (34).

The decrease in NH_3_-N concentration at 0.5% EB is consistent with Garillo et al. (35) who also indicated that NH_3_-N was lower when 0.6% activated carbon was included in a high roughage diet fed to mature goats. Although Garillo et al. (35) reported a change in NH_3_-N production, protozoal counts did not differ among treatments. In contrast, Saleem et al. (1) reported that NH_3_-N tended (*P* = 0.06) to linearly increase with increasing biochar concentration, but it did not alter protozoal populations. The reduction in protozoa observed in the present study has been hypothesized to be a result of the non-selective adsorptive properties of EB (36), but the exact cellular mechanisms whereby such an interaction would lead to a reduction in protozoa have yet to be elucidated. Although there were declines in NH_3_-N and isobutyrate concentrations may have been linked to the EB—mediated decline in protozoa, this response did not alter MP synthesis or OM digestibility.

Protozoa can predate on bacteria that utilize NH_3_-N as a protein source, such that a decrease in protozoal numbers may improve ruminal N metabolism via enriched bacterial protein synthesis (37). However, there were no associated benefits on apparent nutrient digestibility observed in the present study. This finding is consistent with Winders et al. (10) who observed no changes in the total tract digestibility of steers fed either a high forage or high concentrate diet containing biochar at 3% of DM. Counter to this lack of effect, Van et al. (38) found that digestibility of DM, OM, and CP were improved, and N retention increased in growing goats fed 0.5 and 1 g/kg BW of bamboo charcoal. One component which may explain the variation among studies is most likely related to the tannin containing *Acacia mangium* on which the goats used by Van et al. (38) were fed. Biochar can absorb toxic compounds, preventing their absorption from the gastrointestinal tract (39).

The increased minimum pH and decreased variation of pH at 2.0% EB is likely due to the alkalinity (pH 7-8) of the EB used in this study. Van et al. (38) reported that ruminal pH tended to be higher in goats fed activated charcoal. Additionally, Calvelo Pereira et al. (31) reported that grass ensiled with biochar at 186 g/kg DM increased pH after 128 days. The pH of the biochar used by Calvelo Pereira et al. (31) ranged from 9.16 to 9.89 and was produced from either pine chips or corn stover pyrolysed at either 350 or 550°C. The EB used in the present study had a pH of 7-8 due to the neutralization process and as a result would be expected to only minimally increase rumen pH. Comparably, Saleem et al. (1) used a biochar with a pH of 4.8 which did not affect the pH within a RUSITEC.

Though enteric CH_4_ emissions were not affected by EB in the present study, Leng et al. (8) reported that rice husk biochar decreased CH_4_ production by 24.3% and increased live weight gain by 20.2% in young beef cattle. Similarly, Saleem et al. (1) reported a 25.2% reduction in CH_4_ production in the RUSITEC. A number of hypothesis have been proposed as to how biochar may promote a decrease in ruminal CH_4_ emissions. These include the proposal that the high surface area, porous structure, high ion exchange and absorption properties promote the formation of biofilms (9, 40). However methanogens are known to be integral members of biofilms in the rumen (41) and theoretically an increase in biofilm development could actually result in an increase in CH_4_ production in the rumen. Furthermore, based on electron microscopy, surface biofilms formed on biochar (unpublished data, 2019) appear to be much less developed than those formed on the surface of readily digested feeds such as grains (42).

It has also been proposed that biochar may increase the abundance of methanotrophs, inhibit methanogens, or absorb CH_4_ produced within the rumen. However, this has yet to be evaluated. Methanotrophs are capable of anaerobically oxidizing CH_4_ in reactions coupled to the reduction of sulfate, nitrate or metal oxides. The existence and relative importance of methanotrophs within the rumen remains controversial as studies have found them to be absent (43) or only present in the rumen at extremely low abundance (44–47). It is also possible that methanotrophs are deriving energy from metabolic pathways other than that associated with the oxidation of CH_4_. Furthermore, the likelihood that the low concentrations (0.3–3.0% DM) of biochar in the diet could absorb the large amount of CH_4_ produced within the rumen seems improbable. Concurrently, there was no effect of EB on the CH_4_ producing microbes, *Archaea*, at the phylum, family or genera level in SAM and LAM, suggesting that at the concentrations administered, EB also was not directly toxic to methanogens. In agreement with our study, Winders et al. (10) found that whole pine tree biochar fed at either 0.8 or 3.0% of DM did not decrease CH_4_ production in steers fed either a growing or finishing diet.

Although α-diversity indices were not changed by EB, it did cause shifts in several phyla and families. *Fibrobacteres* are recognized as major lignocellulosic degraders within the rumen (48) and their abundance was decreased in both LAM and SAM by EB. *Spirochaetes*, another phylum assumed to play a role in complex fiber degradation (49) was increased by EB in both LAM and SAM. Interestingly, all other phyla that exhibited a response to EB treatment were liquid associated, possibly a reflection of the dispersion of fine EB particles within the liquid fraction of rumen contents. *Verrucomicrobia, Tenericutes*, and *Elusimicrobia* are typically minor members within the rumen microbiome (50) and their role in the rumen remains unclear. *Prevotellaceae*, members of the *Bacteroidetes*, was the dominant family within LAM and were increased by EB. Similarly, two other families of *Bacteroidetes, Bacteroidales* RF16 group and Bacteroidales BS11 gut group were increased in LAM by EB. *Acidaminococcaceae*, belonging to the *Firmicutes*, was decreased by EB and this family was represented by the genus *Succiniclasticum*, which are known to ferment succinate to propionate (50). *Bifidobacteriaceae* belonging to *Actinobacteria* phylum were reduced by 64.0% in both LAM and SAM by EB. This family has been associated with an increased abundance in more fibrous diets and may be involved in metabolizing plant derived complex carbohydrates (50, 51). Despite alterations to these microbial populations, it seems that these were not linked to quantifiable changes in rumen fermentation or metabolism.

In soils, addition of biochar at 30 Mg ha^−1^ was shown to not alter biota (bacteria, fungi, protozoa, nematodes and arthropods) 1 year post-application (52). Additionally, biochar produced by slow-pyrolysis (0.5% soil DM; 15 t/ha) did not alter microbial mass or community structure, however, the abundance of fungi was increased after 720 h of incubation (53). This was possibly due to the ability of fungi to degrade recalcitrant feeds (54). Alternatively, a meta-analysis study found that biochar amended soils increased microbial abundance and diversity after both short and long term application (55). Due to the comparably short residence time in the rumen compared to soils, differences in microbial outcomes are expected.

Further studies are required to estimate the extent to which EB acts within the rumen microbiome. Liquid associated microbes were overall, more impacted by EB than SAM, suggesting that LAM can get passively get trapped in the porous structure of the EB. Alternatively, SAM are already tightly associated with feed particles and are not directly exposed to EB. Despite a notable change in color of feces as a result of inclusion of EB in the diet, there was no change in FAM bacterial populations. Interestingly, there was a tendency for an increase in *Euryarchaeota* in FAM, a phylum associated with CH_4_ producing archaea, despite reports that biochar amended manure decreases CH_4_ emissions during composting (56).

In conclusion, EB did not alter rumen fermentation, CH_4_ production or apparent total tract digestibility.

## Data Availability

The datasets generated for this study are available on request to the corresponding author. Microbial datasets generated for this study can be found in the Small Reads Archive (NCBI) with accession number PRJNA534330.

## Ethics Statement

The animal study was reviewed and approved by this study was conducted at the Agriculture and Agri-Food Canada Research and Development Centre in Lethbridge, Alberta, Canada. The heifers were cared for in accordance with the guidelines of the Canadian Council on Animal Care (11). The study procedures for animal use were reviewed and approved by the institutional Animal Care Committee at the research center (ACC1726).

## Author Contributions

ST, GR, RG, AC, KB, EO, and TM: study design. ST: sample acquisition. ST and RG: lab analysis. ST and AC: statistical analysis. ST and RG: manuscript draft. All authors read and approved the final manuscript.

### Conflict of Interest Statement

The authors declare that the research was conducted in the absence of any commercial or financial relationships that could be construed as a potential conflict of interest.
